# Use of targeted next generation sequencing to characterize tumor mutational burden and efficacy of immune checkpoint inhibition in small cell lung cancer

**DOI:** 10.1186/s40425-019-0572-6

**Published:** 2019-03-28

**Authors:** Biagio Ricciuti, Sasha Kravets, Suzanne E. Dahlberg, Renato Umeton, Adem Albayrak, Safiya J. Subegdjo, Bruce E. Johnson, Mizuki Nishino, Lynette M. Sholl, Mark M. Awad

**Affiliations:** 1 Lowe Center for Thoracic Oncology, Dana-Farber Cancer Institute, Harvard Medical School, 450 Brookline Avenue, Boston, MA 02215 USA; 2 Department of Data Sciences, Division of Biostatistics, Dana-Farber Cancer Institute, Boston, MA USA; 3 Department of Informatics, Dana-Farber Cancer Institute, Boston, MA USA; 4 Massachusetts Institute of Technology, Cambridge, MA USA; 5 Department of Radiology, Brigham and Women’s Hospital and Dana-Farber Cancer Institute, Harvard Medical School, Boston, MA USA; 6 Department of Pathology, Brigham and Women’s Hospital, Boston, Harvard Medical School, Boston, MA USA

**Keywords:** Tumor mutational burden, Immunotherapy, SCLC

## Abstract

**Background:**

Clinically-available biomarkers to identify the fraction of patients with small cell lung cancer (SCLC) who respond to immune-checkpoint inhibitors (ICIs) are lacking. High nonsynonymous tumor mutational burden (TMB), as assessed by whole exome sequencing, correlates with improved clinical outcomes for patients with SCLC treated with ICIs. Whether TMB as assessed by targeted next generation sequencing (NGS) is associated with improved efficacy of ICIs in patients with SCLC is currently unknown. Here we determined whether TMB by targeted NGS is associated with efficacy of ICIs in patients with SCLC.

**Methods:**

We collected clinicopathologic data from patients with relapsed or refractory SCLC which underwent targeted NGS with TMB assessment by the Dana-Farber Cancer Institute OncoPanel platform. The relationship between TMB and clinical outcomes after treatment with ICIs was investigated.

**Results:**

Among the 52 patients treated with ICIs, we found no significant difference in the objective response rate (ORR) between patients with a TMB above the 50th percentile (“TMB high”) and those with a TMB at or below the 50th percentile (“TMB low”). The median progression-free survival (mPFS) and median overall survival (mOS) were significantly longer in patients with a high TMB compared to those with a low TMB (mPFS: 3.3 versus 1.2 months, HR: 0.37 [95% CI: 0.20–0.69], *P* < 0.01; mOS: 10.4 versus 2.5 months, HR: 0.38 [95% CI: 0.19–0.77], *P* < 0.01). The one-year PFS and OS rates improved with increasing mutational load when TMB was divided into tertiles.

**Conclusions:**

These findings show that targeted NGS, a readily available clinical diagnostic test, can be used to identify patients with SCLC who are most likely to benefit from treatment with immune checkpoint inhibitors.

**Electronic supplementary material:**

The online version of this article (10.1186/s40425-019-0572-6) contains supplementary material, which is available to authorized users.

## Introduction

Although the majority of patients diagnosed with extensive-stage small cell lung cancer (ES-SCLC) respond to first-line chemotherapy, relapse invariably occurs and only 5% of patients are alive two years after initial diagnosis [1–3]. In the past several decades, very little progress has been made in developing effective systemic therapies for SCLC [4]. Programmed death (PD)-1 inhibitors, either alone or in combination with cytotoxic T-cell lymphocyte 4 (CTLA-4) inhibitors have shown promising antitumor activity in a subset of patients with previously-treated SCLC. In the CheckMate 032 phase I/II trial [5], the objective response rate (ORR) to nivolumab monotherapy and nivolumab plus ipilimumab was 11 and 23%, and the two-year overall survival rates were 14 and 26%, respectively [6]. Based on these results, single-agent nivolumab was granted accelerated FDA approval for patients with SCLC with disease progression following platinum-based chemotherapy and one other line of therapy. Additionally, among 24 patients with PD-L1 positive SCLC treated with the PD-1 inhibitor pembrolizumab in the KEYNOTE-028 phase 1b study, the ORR was 33% [7]. Recently, the phase I/III IMpower 133 trial demonstrated an overall survival benefit when the PD-L1 inhibitor atezolizumab was added to platinum/etoposide chemotherapy for the initial treatment of ES-SCLC [8], although why only a subset of patients benefitted from this combination therapy is not currently known.

Unfortunately, the identification of predictive biomarkers of efficacy of immune checkpoint inhibitors (ICIs) in SCLC has been challenging. In contrast to the ~ 60% of non-small cell lung cancers (NSCLCs) which are positive for expression of the programmed death ligand 1 (PD-L1) [9], only approximately 18–32% of SCLC cases are PD-L1 positive [5, 7]. Furthermore, responses to nivolumab alone or in combination with ipilimumab do not appear to correlate with PD-L1 expression, which argues against the use of PD-L1 as predictive biomarker for immunotherapy in SCLC [5, 6], and highlights the need to identify novel biomarkers in this disease.

In several tumor types, such as NSCLC, melanoma, and urothelial carcinomas, cancers with a high number of non-synonymous somatic mutations, and therefore a greater neoantigen load which be recognized and targeted by immune cells tend to have higher response rates to immune checkpoint inhibitors than cancers with a low tumor mutational burden (TMB) [10–16]. Although mechanisms underlying the association between TMB and benefit from ICIs are not fully understood, tumor-specific neoantigens resulting from somatic nonsynonymous mutations may elicit neoantigen-specific T-cell responses that direct anti-tumor immunity [17]. SCLC, which is almost invariably associated with smoking, has among the highest mutational loads across cancer types, likely owing to tobacco-induced mutagenesis, which is characterized by a high transversion/transition ratio and increased genomic instability [18–21]. A recent exploratory analysis of the CheckMate 032 study using whole exome sequencing (WES) with paired germline sequencing to quantify tumor somatic mutational load found that the estimated one-year progression-free survival rates were higher in the high TMB group (21.2 and 30.0% for nivolumab monotherapy and nivolumab plus ipilimumab, respectively) compared with the low (not calculable and 6.2%, respectively) or medium (3.1 and 8.0%, respectively) TMB groups. Similarly, within each treatment group, the estimated one-year overall survival rate was higher in the high TMB group (35.2 and 62.4% for nivolumab monotherapy and nivolumab plus ipilimumab, respectively) than in the low (22.1 and 23.4%, respectively) or medium (26.0 and 19.6%, respectively) tumor mutational burden groups [22]. By contrast, exploratory subgroup analyses of the IMpower 133 showed no clear suggestion that blood-based TMB is associated with clinical outcome in patients receiving chemotherapy plus atezolizumab [8].

While WES may be the best-established technique for quantifying mutations in the coding genome, this technique is not readily available to most practicing clinicians since it requires significant informatics expertise and relies on sequencing of paired normal samples to filter out germline variants. Targeted next generation sequencing (NGS) is a relatively fast, cost-effective, clinically-available tool for estimating TMB, and there is generally good correlation between NGS and WES for determining TMB [23–26]. Whether TMB as assessed by targeted NGS is associated with improved efficacy of ICIs in patients with advanced SCLC is still unknown.

In the present study we investigate the feasibility of using targeted NGS to quantify TMB in SCLC and determine if patients with SCLC and a high TMB are more likely to benefit from treatment with immune checkpoint inhibitors than in patients with SCLC and a low TMB.

## Methods

### Study population

We retrospectively collected clinicopathologic data from patients with relapsed or refractory SCLC who had consented to a correlative research study (DF/HCC protocol #02–180). Patients were included if their tumors underwent successful targeted NGS between July 2014 and July 2018, at the Dana-Farber Cancer Institute (DFCI). The immunotherapy-treated cohort included patients who were treated with PD-1 and/or CTLA-4 inhibitors. Tumor mutational burden (TMB), defined as the number of somatic, coding, base substitution and indel mutations per megabase (Mb) of genome examined was calculated from the DFCI OncoPanel NGS platforms as previously described [26].

### Clinical outcomes

To determine ORR and progression-free survival (PFS), scans were reviewed by a dedicated thoracic oncologist using Response Evaluation Criteria In Solid Tumors (RECIST) version 1.1.

Progression-free survival (PFS) was defined as the time from the start of immunotherapy or chemotherapy to the date of disease progression or death, whichever occurred first. Patients who were alive without disease progression were censored on the date of their last adequate disease assessment. Overall survival (OS) was defined as the time from the start of immunotherapy to death. Patients who were still alive were censored at the date of last contact. As a complementary analysis, OS was also calculated from the date of initial pathologic SCLC diagnosis. To validate the predictive nature of TMB in patients with SCLC treated with ICIs, survival outcomes were also evaluated in a cohort of patients who never received ICIs.

### Statistical analysis

Categorical and continuous variables were summarized descriptively using percentages and medians. The Wilcoxon-Rank Sum test and Kruskal-Wallis test were used to test for differences between continuous variables, and Fisher’s exact test was used to test for associations between categorical variables. Kaplan-Meier methodology was used to estimate event-time distributions, and the Greenwood formula was used to estimate the standard errors of the estimates. Log-rank tests were used to test for differences in event-time distributions, and Cox proportional hazards models were fitted to obtain estimates of hazard ratios in univariate and multivariable models. All *p*-values are two-sided and confidence intervals are at the 95% level, with statistical significance defined as *P* ≤ 0.05.

## Results

### Patient characteristics and tumor mutational burden

Of 134 SCLCs which underwent successful targeted NGS with TMB assessment, 52 (38.8%) were treated with ICIs (Additional file 1: Figure S1), and 82 (61.2%) did not receive ICIs for the following reasons: 21 never received any systemic therapy due to poor performance status or because their cancer had not recurred after definitive treatment for limited-stage SCLC; 49 did not receive ICIs because they received treatment between March 2012 and May 2018 prior to the FDA approval of immunotherapy for SCLC and were not able to get immunotherapy on clinical trials; 12 had not progressed on their last systemic treatment prior to the data cut-off. In the ICI-treated cohort, 31 (59.6%) received anti-PD-1 monotherapy (24 received nivolumab; 7 received pembrolizumab) and 21 (40.4%) received nivolumab in combination with ipilimumab. Immunotherapy was administered in the setting of a clinical trial in 22 (42.3%) patients, and 30 patients (57.7%) received commercial immunotherapy. The median age of patients was 65 (range: 43–84) and 94.2% were current or former smokers. In the entire cohort of 134 TMB-evaluable SCLC patients, the median TMB was 9.68 mutations/megabase (mut/Mb) (range: 1.21–31.18), and a similar TMB distribution was observed in the subgroup of 52 ICI-treated patients (median: 9.78, range: 1.33–31.18, Additional file 2: Figure S2). Targeted NGS was performed in all cases on tumor specimens obtained at the time of initial pathologic diagnosis. “TMB high” was defined as cases with a TMB above the 50th percentile (TMB > 9.68 mut/Mb), and “TMB low” was defined as cases at or below the 50th percentile (≤ 9.68 mut/Mb). Baseline clinicopathological characteristics were balanced between the TMB high and TMB low groups, as summarized in Table 1. TMB was also analyzed in tertiles: “TMB upper” (> 12.10 mut/Mb), “TMB middle” (between 12.10 and 8.36 mut/Mb, inclusive), and “TMB lower” (< 8.36 mut/Mb).

**Table 1:** Baseline clinicopathologic characteristics of patients

		Total *N* = 52 (%)	TMB high (> 9.68 mut/Mb) *N* = 26 (%)	TMB low (≤ 9.68 mut/Mb) *N* = 26 (%)	*P*-value^a^
Age	Median	65	63.5	67.5	0.18
	Range	43–84	47–84	43–83	
Sex	Male	25 (48.1)	10 (38.5)	15 (57.7)	0.27
	Female	27 (51.9)	16 (61.5)	11 (42.3)	
Smoking Status	Current/Former	49 (94.2)	26 (100)	23 (88.5)	0.24
	Never	3 (5.8)	0 (0.0)	3 (11.5)	
EGFR status	Mutant	3 (5.8)	0 (0.0)	3 (11.5)	0.24
	Wild type	49 (94.2)	26 (100)	23 (88.5)	
Stage at Diagnosis	Extensive	34 (65.4)	15 (57.7)	19 (73.1)	0.38
	Limited	18 (34.6)	11 (42.3)	7 (26.9)	
ECOG PS	0	7 (13.5)	3 (11.5)	4 (15.4)	0.24^b^
	1	28 (53.8)	17 (65.4)	11 (42.3)	
	2	15 (28.8)	5 (19.2)	10 (38.5)	
	3	2 (3.8)	1 (3.8)	1 (3.8)	
Response to platinum doublet	Platinum sensitive	26 (50.0)	15 (57.7)	11 (42.3)	0.41^c^
	Platinum resistant	19 (36.5)	10 (38.5)	9 (34.6)	
	Platinum refractory	7 (13.5)	1 (3.8)	6 (23.1)	
Treatment received	PD-1-monotherapy^d^	31 (59.6)	17 (65.4)	14 (53.8)	0.57
	PD-1 + CTLA-4	21 (40.4)	9 (34.6)	12 (46.2)	
Treatment setting	Clinical trial	22 (42.3)	14 (53.8)	8 (30.8)	0.16
	Commercial	30 (57.7)	12 (46.2)	18 (69.2)	
Line of therapy	2	29 (55.8)	18 (69.2)	11 (42.3)	0.09^e^
	3	15 (28.8)	6 (23.1)	9 (34.6)	
	≥ 4	8 (15.4)	2 (7.7)	6 (23.1)	
Brain metastasis prior to immunotherapy	Yes	17 (32.7)	7 (26.9)	10 (38.5)	0.56
	No	35 (67.3)	19 (73.1)	16 (61.5)	

Abbreviations: *ECOG PS* Eastern Cooperative Oncology Group Performance Status, *EGFR* Epidermal growth factor receptor

^a^P values are comparing TMB high and TMB low columns

^b^ECOG PS: 0–1 vs ≥ 2

^c^Platinum sensitivity: platinum sensitive vs platinum resistant/refractory

^d^One patient received anti PD-1 agent pembrolizumab in combination with a PIK3CA inhibitor; the remainder of patients received PD-1 monotherapy

^e^Line of therapy: 2 vs ≥ 2

### Association between TMB and efficacy of immunotherapy

In the cohort of 52 TMB-evaluable and ICI-treated SCLC patients, the objective response rate (ORR) was 15.4% (95% CI: 6.9–28.1%), and the disease control rate (DCR) was 38.5% (95% CI: 25.3–53.0%). With a median follow-up of 24.9 months (95% CI: 15.9-NR), the median PFS (mPFS) was 1.7 months (95% CI: 1.3–2.4), and the median OS (mOS) was 5.9 months (95% CI: 2.7–13.2), Additional file 3: Figure S3 A-B, calculated from the start date of immunotherapy.

**Fig. 1:**
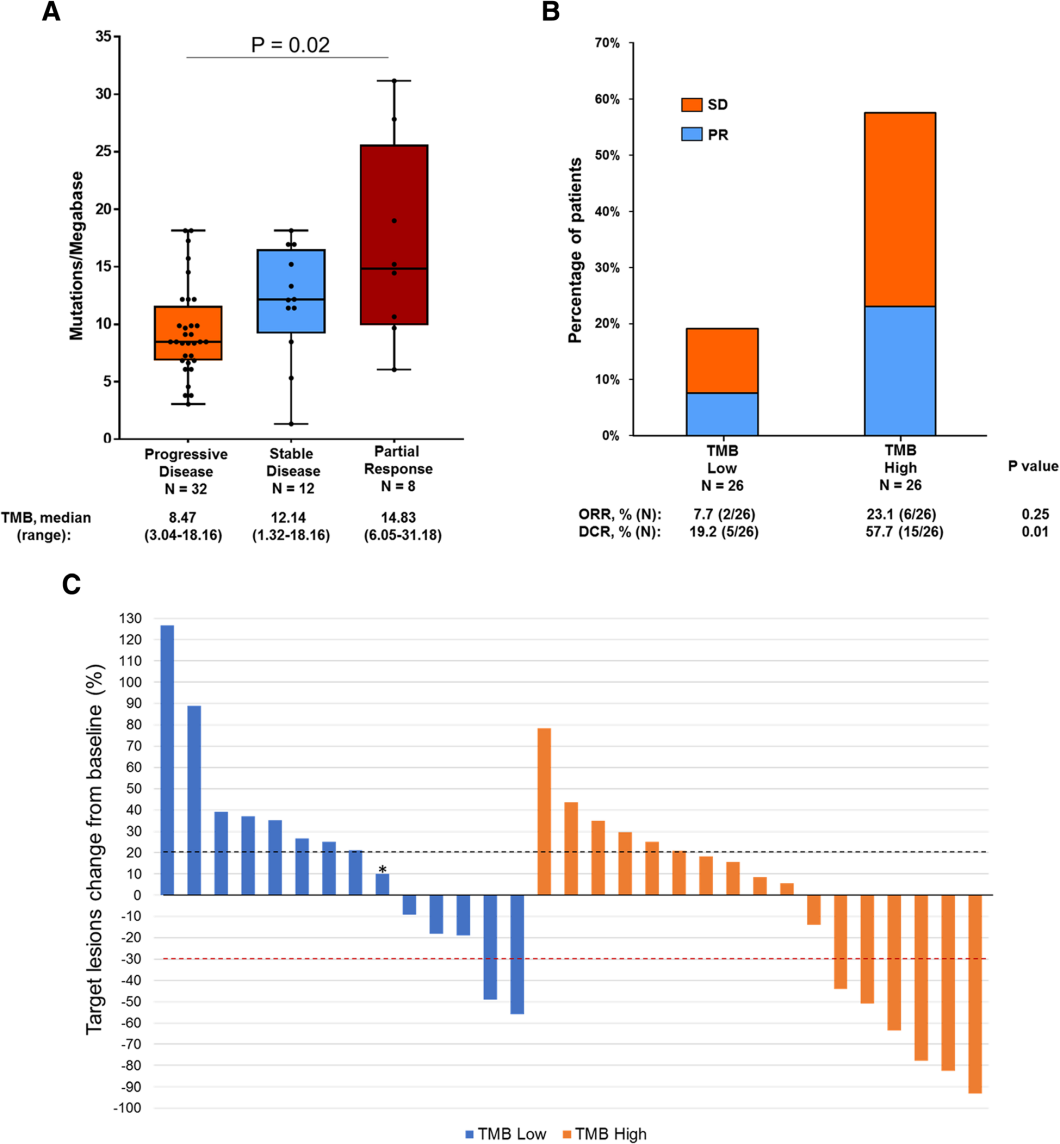
**a** Tumor mutational burden (TMB) in patients who had a partial response (PR), stable disease (SD), or primary progressive disease (PD). Box plots represent medians, interquartile ranges, and vertical lines extend to the highest and the lowest TMB values. TMB of individual patients are represented with dots. **b** Proportion of patients with PR and SD in the TMB high versus TMB low groups (**c**) Waterfall plot showing the change (%) of tumor burden compared to baseline in patients with evaluable target lesions (*N* = 31). Among non-evaluable patients, 17 had clinical progression and died before scans while 4 had non measurable disease. One patient (indicated with an asterisk) had progressive disease in a non-target lesion

We next sought to investigate the association between TMB and clinical benefit from ICIs. Overall there was a significant difference in TMB between patients who experienced a partial response, stable disease, and progressive disease (*P* = 0.02, Fig. 1a). Patients who experienced a partial response (PR) as their best objective response (BOR) to immunotherapy had a higher median TMB compared to those who had progressive disease (PD) as their BOR (14.83 versus 8.47 mut/Mb). When grouped together, patients who achieved either a PR or stable disease (SD) as their BOR had a significantly higher median TMB compared to those who had PD as their BOR (12.74 versus 8.47 mut/Mb, *P* < 0.01, Additional file 4: Figure S4). Although there was no significant difference in the ORR between patients in the TMB high group (6 of 26, 23.1%) and the TMB low group (2 of 26, 7.7%, *P* = 0.25) (Fig. 1b), TMB high patients had a significantly higher DCR compared to TMB low patients (57.7% versus 19.2%, *P* = 0.01).

**Fig. 2:**
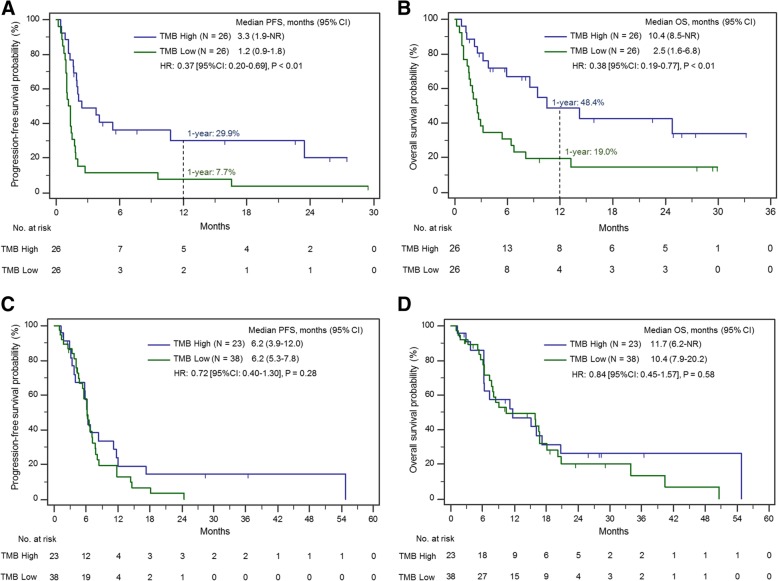
Progression-free (**a**) and overall (**b**) survival in patients treated with immunotherapy in the TMB high and TMB low cohorts, calculated from the start of immunotherapy. Progression-free (**c**) and overall (**d**) survival among patients with ES-SCLC who never received immunotherapy according to TMB status, calculated from the start date of first-line platinum/etoposide chemotherapy

We next examined the progression-free and overall survival according to TMB. The mPFS was significantly longer in the TMB high group compared to the TMB low group (3.3 versus 1.2 months, HR: 0.37 [95% CI: 0.20–0.69], *P* < 0.01, Fig. 2a). In addition, mOS was significantly longer in the TMB high group compared to the TMB low group, whether calculated from the start date of immunotherapy (10.4 versus 2.5 months, HR: 0.38 [95% CI: 0.19–0.77], P < 0.01, Fig. 2b) or from the date of initial SCLC pathologic diagnosis (33.9 versus 15.6 months, HR: 0.39 [95% CI 0.19–0.79], P < 0.01, Additional file 5: Figure S5). Importantly, in a univariate model, we found that gender, baseline brain metastases and type of treatment received (anti PD-1 + anti CTLA-4 versus anti PD-1 monotherapy), were not significantly associated with OS. However, both age (< 70 versus ≥ 70 years, HR: 0.44 [95% CI: 0.22–0.87], *P* = 0.02) and Eastern Cooperative Oncology Group performance status (ECOG-PS) (ECOG 0–1 versus ≥2, HR: 0.44 [95% CI: 0.22–0.88, *P* = 0.02) were significantly associated with OS. We then ran a multivariate model with TMB, adjusting for age and ECOG PS, to evaluate whether TMB was still significantly associated with OS. After adjusting for age (< 70 versus ≥70 years, HR: 0.59 [0.28–1.28], *P* = 0.1801), Eastern Cooperative Oncology Group performance status (ECOG-PS) (ECOG 0–1 versus ≥2, HR: 0.66 [0.30–1.46], *P* = 0.31) we found that a TMB above median retained a significant association with a longer OS in multivariable analysis (HR: 0.47 [95% CI: 0.22–0.97], *P* = 0.04). In light of the continuous nature of TMB as variable, we also performed a univariate Cox model with TMB as a continuous variable and found that TMB maintains its significant association with both prolonged PFS (HR: 0.91 [95% CI: 0.85–0.96], *P* < 0.01) and OS (HR: 0.89 [95% CI: 0.83–0.96], *P* < 0.01).

To further confirm that TMB is a predictive biomarker only for immunotherapy and not for chemotherapy, we next examined the relationship between TMB and clinical outcomes with chemotherapy. Among the 61 patients with ES-SCLC treated with first-line platinum/etoposide who never received subsequent immunotherapy, there was no association between TMB and mPFS (6.2 versus 6.2 months, HR: 0.72 [95%CI: 0.40–1.30], *P* = 0.28) or mOS (11.7 versus 10.4 months, HR: 0.84 [95% CI: 0.45–1.57], *P* = 0.58) when calculated from the start date of first-line chemotherapy (Fig. 2 c-d). Similarly, among the 52 ICI-treated patients, there was no significant difference in mPFS to first-line platinum/etoposide between the TMB high and TMB low groups (6.2 versus 5.6 months, HR: 0.59 [95% CI: 0.34–1.04], *P* = 0.07, Additional file 6: Figure S6). Lastly we performed a Cox model with an interaction between TMB as a continuous measure and whether or not the patient received immunotherapy. We found that the effect of higher TMB on prolonged overall survival was restricted tothose patients who received immunotherapy, but did not impact survival in patients who never received immunotherapy (*P* = 0.04).

**Fig. 3:**
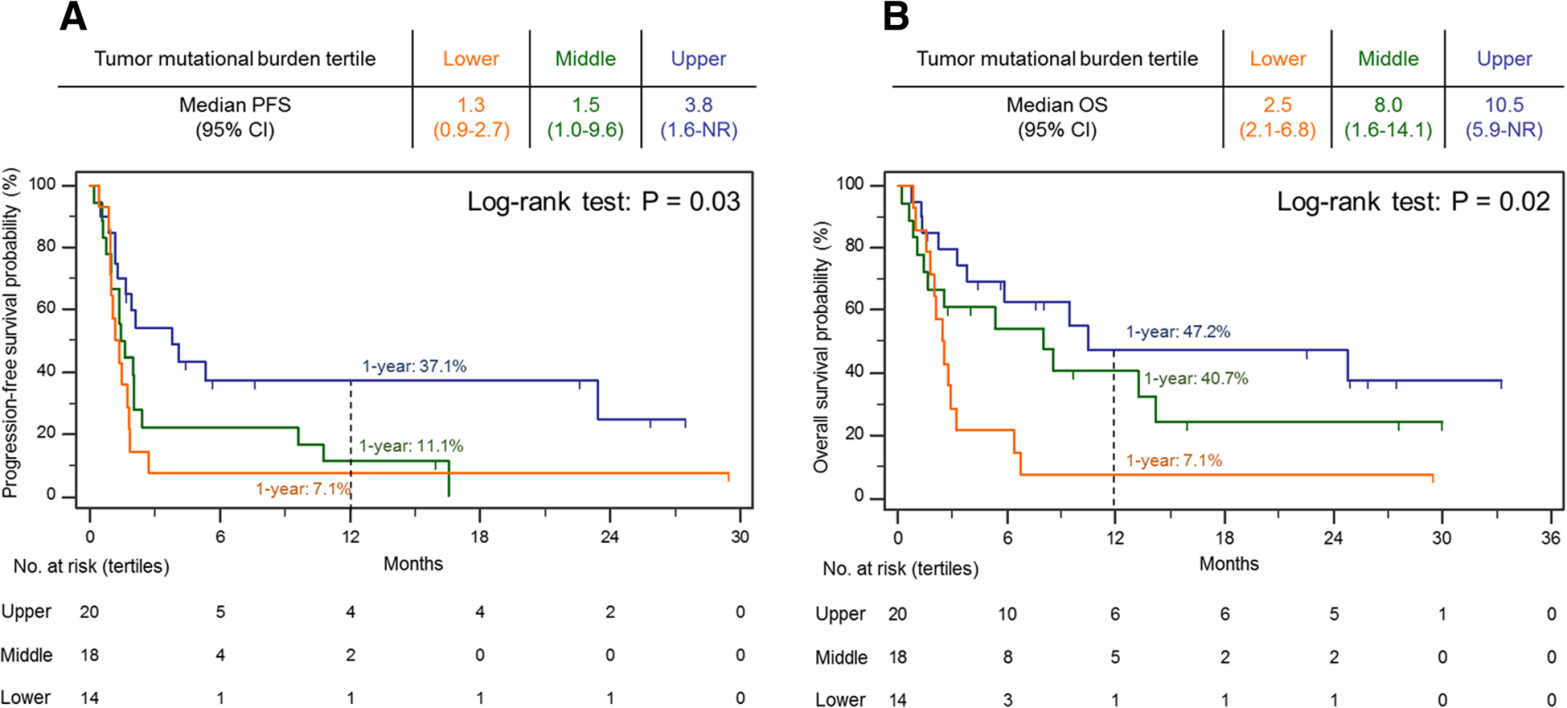
Progression-free (**a**) and overall (**b**) survival by tumor mutational burden (TMB) tertiles

We next investigated clinical outcomes when SCLCs were stratified by increasing TMB tertiles. We found the mPFS (95% CI) increased from 1.3 (0.9–2.7) to 1.5 (1.0–9.6) to 3.8 (1.6-NR) months, in the lower, middle, and upper tertiles, respectively (*P* = 0.03), and the mOS (95% CI) increased from 2.5 (2.1–6.8) to 8.0 (1.6–14.1) to 10.5 (5.9-NR) months in the lower, middle, and upper tertiles, respectively (*P* = 0.02). Consistently, the 1-year survival rates increased along with increasing TMB cutoffs. The 1-year PFS rate was 7.1, 11.1 and 37.1% in the lower, middle, and upper tertiles, respectively, and the 1-year OS rate was 7.1, 40.7, 47.2% in the lower, middle, and upper tertiles, respectively (Fig. 3 a-b).

## Discussion

Although ICIs can provide a substantial clinical benefit in a small proportion of patients with SCLC, the lack of clinically-accessible predictive biomarkers makes it challenging to identify patients who are more likely to respond to ICIs. Recent evidence using WES with paired germline sequencing has shown that high-TMB SCLCs are more likely to benefit from treatment with nivolumab ± ipilimumab [22]. However, whether TMB as assessed by targeted NGS is associated with immunotherapy efficacy in patients with SCLC is unknown. To address this, we conducted a retrospective study using targeted sequencing data to evaluate the impact of TMB on ICI efficacy in a cohort of patients with SCLC.

We found that patients with SCLC and an elevated TMB had significantly better clinical outcomes after immunotherapy treatment compared to those with lower TMB. Highlighting the continuous nature of TMB as a predictive biomarker, we also demonstrated that 1-year PFS and OS rates improved with increasing mutational load when TMB was divided into tertiles. Importantly, supporting the hypothesis that TMB is predictive of immunotherapy benefit, we found no association between TMB and outcomes in patients treated only with chemotherapy. Limitations of this study include that this was a retrospective analysis on a relatively small sample size of patients treated both on clinical trials as well as on commercial immunotherapy, and there was also heterogeneity of treatment with different PD-1 inhibitors with or without combined CTLA-4 inhibition.

In the context of available literature, our data provide the first evidence for the use of targeted NGS to assess TMB status for the prediction of efficacy of ICIs in SCLC. In contrast to WES, TMB can be easily assessed using targeted NGS profiling panels that are already in routine clinical use. Several reports have recently sequenced the same tumors with both WES and targeted NGS and found that TMB determined by WES closely correlated with TMB determined by NGS in different tumor types, including in SCLC [20, 23, 24]. However, not all sequencing panels can accurately estimate TMB, especially those with low genomic coverage < 0.5 Mb [23].

Whether TMB is also predictive in patients with SCLC treated with a combination of chemotherapy plus immunotherapy is unclear. An exploratory subgroup analysis of the IMpower 133 SCLC study of platinum/etoposide ± atezolizumab showed no clear evidence that high blood-based TMB (bTMB) levels were associated with improved clinical outcomes [8], but TMB from tumor tissue was not reported in this study. Other recent analyses have shown that high bTMB may identify patients who derive a clinical benefit from atezolizumab in previously-treated NSCLC [27]. Additional prospective analyses on the role of blood- versus tissue-based mutational load will be needed to identify the optimal technique for biomarker assessment in SCLC and other cancers.

How TMB will be incorporated into clinical decision-making for SCLC at this time is in need of further study, particularly because there is no clearly-established TMB cutoff for patient selection. Given the very limited treatment options currently available for patients with SCLC, immunotherapy should not be withheld from patients with SCLC and a low TMB. As more effective treatment options hopefully become available for patients with SCLC, TMB might be a useful biomarker in determining the order in which therapies are administered. Given the potential for unprecedented, durable responses to ICIs in patients with SCLC, use of targeted NGS to identify high-TMB tumors can rapidly identify patients who should be treated with immunotherapy without delay.

## Additional files

Additional file 1: **Figure S1.** Diagram of patients with SCLC who underwent successful next generation sequencing who either did or did not receive treatment with immune checkpoint inhibitors. Patients who never received any systemic therapy for their disease are indicated. (DOCX 81 kb)

Additional file 2: **Figure S2.** Box plot showing the distribution of TMB between the entire cohort of patients with SCLC and the cohort of patients with SCLC treated with immune checkpoint inhibitors. Box plots represent medians, interquartile ranges, and vertical lines extend to the highest and the lowest TMB values. TMB of individual patients are represented with dots. (DOCX 81 kb)

Additional file 3: **Figure S3.** Kaplan-Meier analysis of (A) progression-free survival (PFS) and (B) overall survival (OS) in the entire cohort of SCLC patients treated with immune checkpoint inhibitors, calculated from the start date of immunotherapy. (DOCX 89 kb)

Additional file 4: **Figure S4.** Box plot showing the distribution of TMB between those who had a partial response (PR) or stable disease (SD) to immunotherapy compared to patients who had primary progressive disease (PD). Box plots represent medians, interquartile ranges, and vertical lines extend to the highest and the lowest TMB values. TMB of individual patients are represented with dots. (DOCX 62 kb)

Additional file 5: **Figure S5.** Kaplan-Meier analysis of overall survival (OS) calculated from the date of initial pathologic diagnosis of SCLC in the immunotherapy-treated cohort. (DOCX 89 kb)

Additional file 6: **Figure S6.** Kaplan-Meier analysis of progression-free survival (PFS) to first-line chemotherapy in the immunotherapy treated cohort. (DOCX 87 kb)

## Abbreviations

CTLA-4: Cytotoxic T-cell lymphocyte antigen 4
ES: Extensive stage
ICI: Immune checkpoint inhibitor
Mb: Megabase
NGS: Next generation sequencing
NSCLC: Non-small cell lung cancer
ORR: Objective response rate
OS: Overall survival
PD-(L)1: Programmed death (ligand) 1
PFS: Progression-free survival
SCLC: Small-cell lung cancer
TMB: Tumor mutational burden
WES: Whole exome sequencing
