# Supplementary material for: Use of targeted next generation sequencing to characterize tumor mutational burden and efficacy of immune checkpoint inhibition in small cell lung cancer
Source: J Immunother Cancer. 2019 Mar 28;7:87. doi: 10.1186/s40425-019-0572-6 (PMC6437848; doi:10.1186/s40425-019-0572-6)
Supplement: Supplementary file 1 — Figure S1. Diagram of patients with SCLC who underwent successful next generation sequencing who either did or did not receive treatment with immune checkpoint inhibitors. Patients who never received any systemic therapy for their disease are indicated. (DOCX 81 kb) [file 40425_2019_572_MOESM1_ESM.docx]

**Figure S1**

**
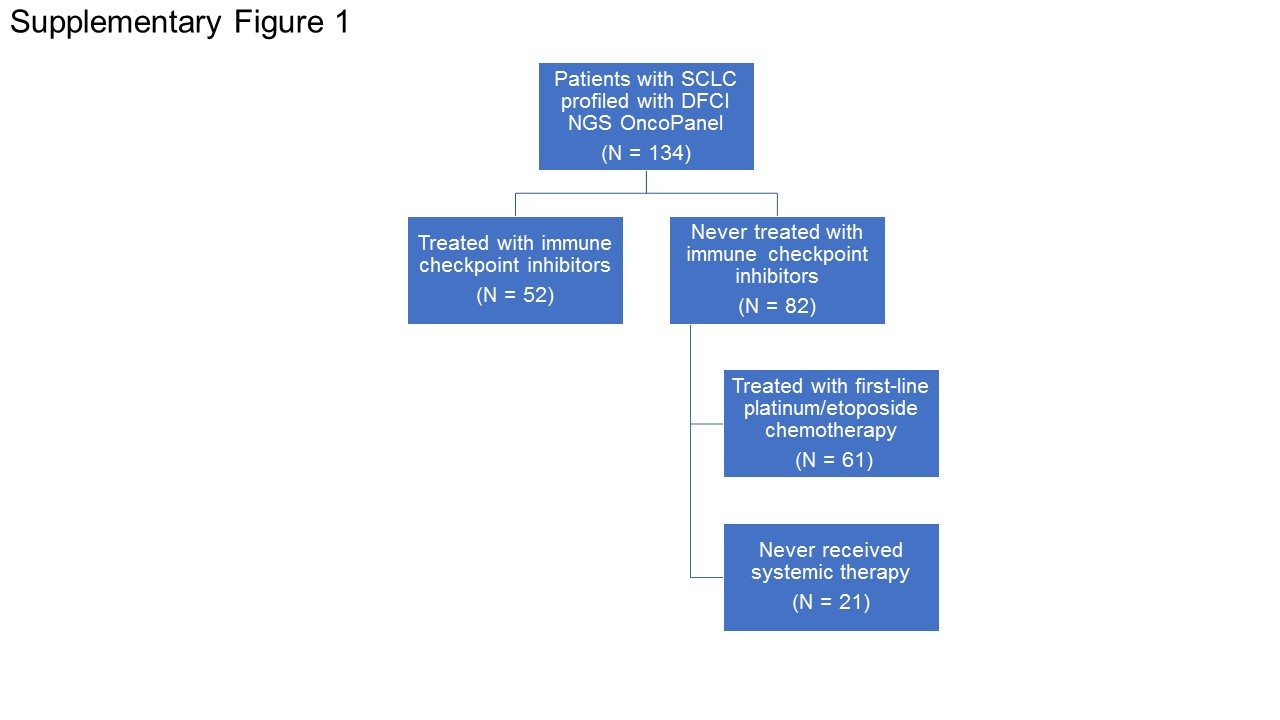
Figure S1.** Diagram of patients with SCLC who underwent successful next generation sequencing who either did or did not receive treatment with immune checkpoint inhibitors. Patients who never received any systemic therapy for their disease are indicated.
