# Supplementary material for: Use of targeted next generation sequencing to characterize tumor mutational burden and efficacy of immune checkpoint inhibition in small cell lung cancer
Source: J Immunother Cancer. 2019 Mar 28;7:87. doi: 10.1186/s40425-019-0572-6 (PMC6437848; doi:10.1186/s40425-019-0572-6)
Supplement: Supplementary file 2 — Figure S2. Box plot showing the distribution of TMB between the entire cohort of patients with SCLC and the cohort of patients with SCLC treated with immune checkpoint inhibitors. Box plots represent medians, interquartile ranges, and vertical lines extend to the highest and the lowest TMB values. TMB of individual patients are represented with dots. (DOCX 81 kb) [file 40425_2019_572_MOESM2_ESM.docx]

**Figure S2
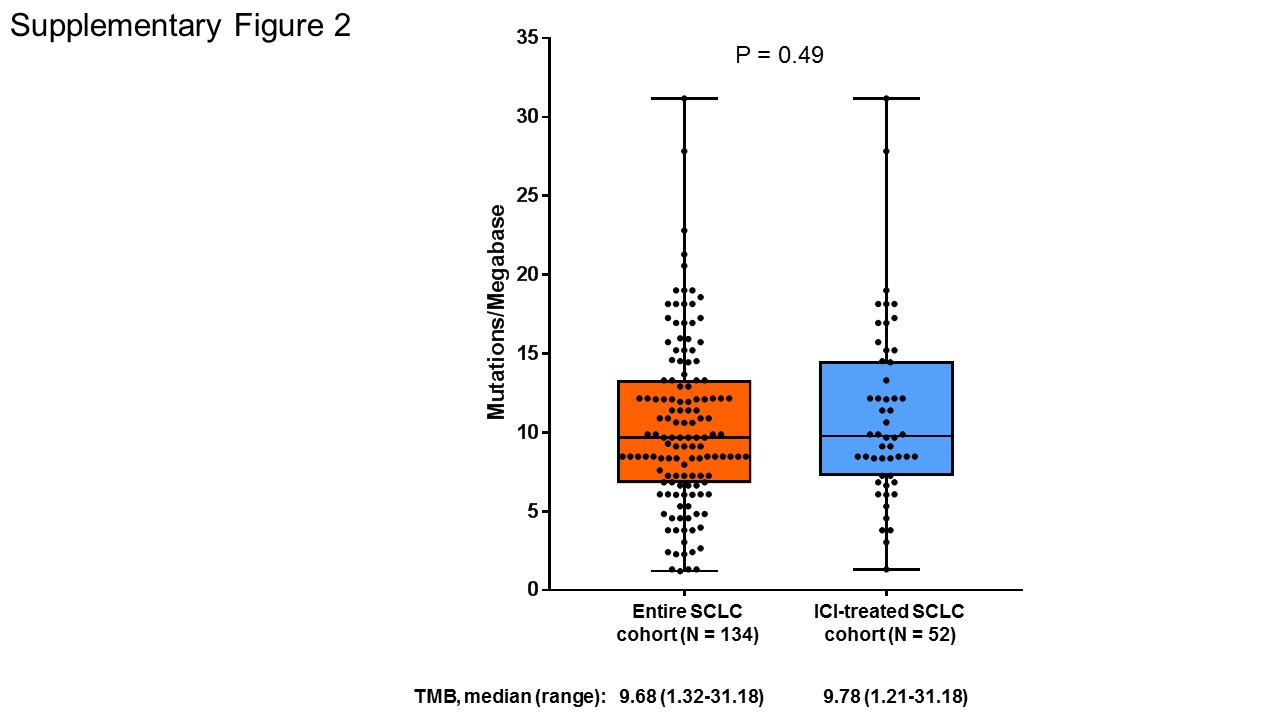
**

**Figure S2.** Box plot showing the distribution of TMB between the entire cohort of patients with SCLC and the cohort of patients with SCLC treated with immune checkpoint inhibitors. Box plots represent medians, interquartile ranges, and vertical lines extend to the highest and the lowest TMB values. TMB of individual patients are represented with dots.
