# Supplementary material for: Use of targeted next generation sequencing to characterize tumor mutational burden and efficacy of immune checkpoint inhibition in small cell lung cancer
Source: J Immunother Cancer. 2019 Mar 28;7:87. doi: 10.1186/s40425-019-0572-6 (PMC6437848; doi:10.1186/s40425-019-0572-6)
Supplement: Supplementary file 3 — Figure S3. Kaplan-Meier analysis of (A) progression-free survival (PFS) and (B) overall survival (OS) in the entire cohort of SCLC patients treated with immune checkpoint inhibitors, calculated from the start date of immunotherapy. (DOCX 89 kb) [file 40425_2019_572_MOESM3_ESM.docx]

**Figure S3**

**
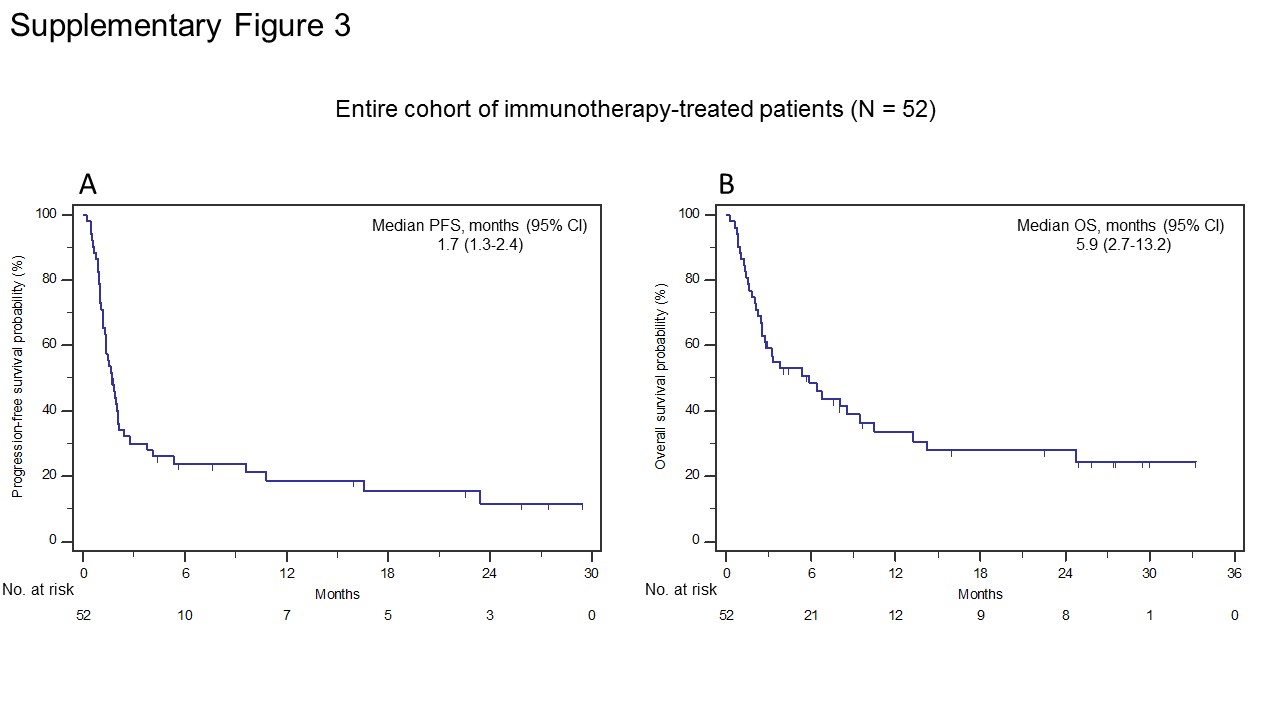
Figure S3.** Kaplan-Meier analysis of **(A)** progression-free survival (PFS) and **(B)** overall survival (OS) in the entire cohort of SCLC patients treated with immune checkpoint inhibitors, calculated from the start date of immunotherapy.
