# Supplementary material for: Use of targeted next generation sequencing to characterize tumor mutational burden and efficacy of immune checkpoint inhibition in small cell lung cancer
Source: J Immunother Cancer. 2019 Mar 28;7:87. doi: 10.1186/s40425-019-0572-6 (PMC6437848; doi:10.1186/s40425-019-0572-6)
Supplement: Supplementary file 4 — Figure S4. Box plot showing the distribution of TMB between those who had a partial response (PR) or stable disease (SD) to immunotherapy compared to patients who had primary progressive disease (PD). Box plots represent medians, interquartile ranges, and vertical lines extend to the highest and the lowest TMB values. TMB of individual patients are represented with dots. (DOCX 62 kb) [file 40425_2019_572_MOESM4_ESM.docx]

**Figure S4**

**
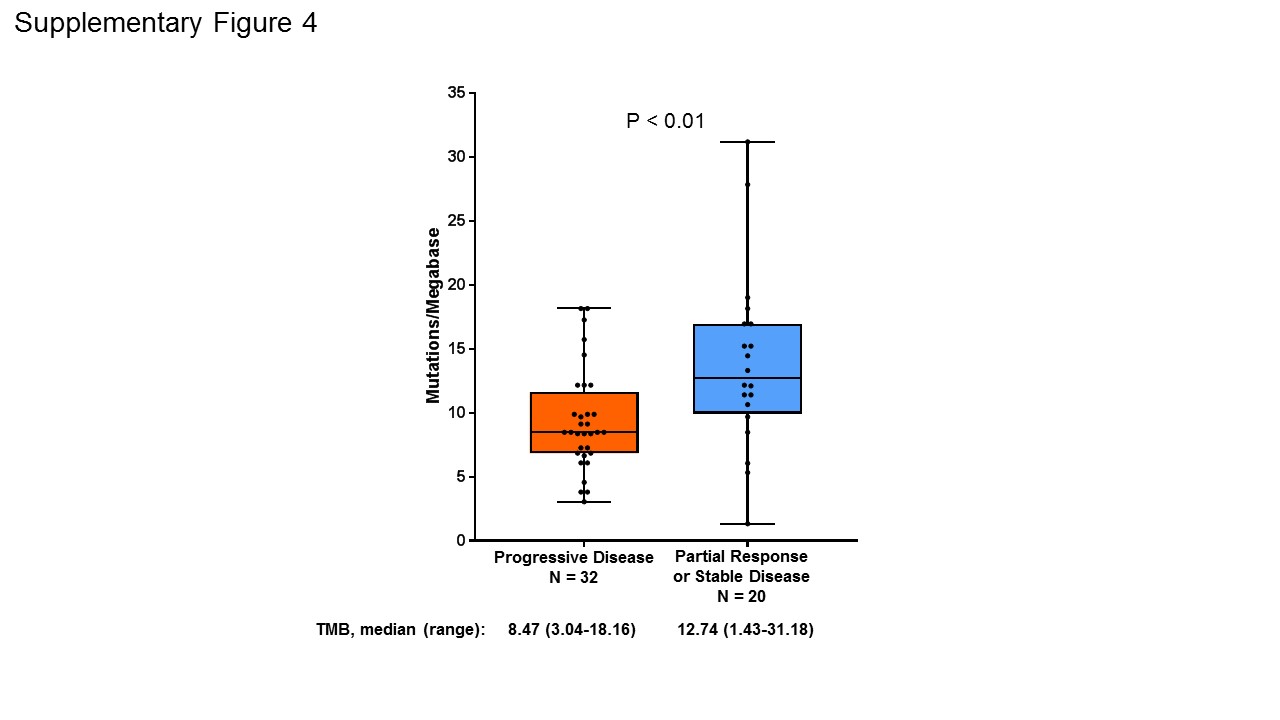
**

**Figure S4.** Box plot showing the distribution of TMB between those who had a partial response (PR) or stable disease (SD) to immunotherapy compared to patients who had primary progressive disease (PD). Box plots represent medians, interquartile ranges, and vertical lines extend to the highest and the lowest TMB values. TMB of individual patients are represented with dots.
