# Supplementary material for: Use of targeted next generation sequencing to characterize tumor mutational burden and efficacy of immune checkpoint inhibition in small cell lung cancer
Source: J Immunother Cancer. 2019 Mar 28;7:87. doi: 10.1186/s40425-019-0572-6 (PMC6437848; doi:10.1186/s40425-019-0572-6)
Supplement: Supplementary file 5 — Figure S5. Kaplan-Meier analysis of overall survival (OS) calculated from the date of initial pathologic diagnosis of SCLC in the immunotherapy-treated cohort. (DOCX 89 kb) [file 40425_2019_572_MOESM5_ESM.docx]

**Figure S5**

**
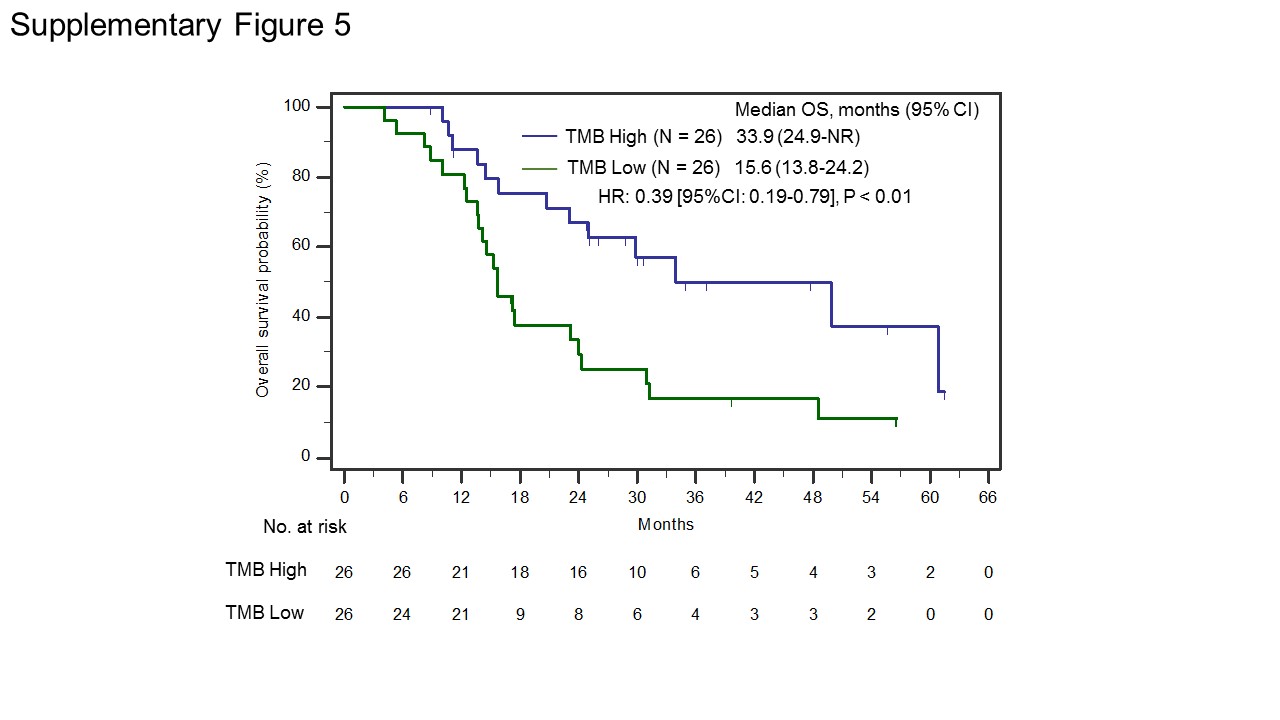
**

**Figure S5.** Kaplan-Meier analysis of overall survival (OS) calculated from the date of initial pathologic diagnosis of SCLC in the immunotherapy-treated cohort.
