# Supplementary material for: Use of targeted next generation sequencing to characterize tumor mutational burden and efficacy of immune checkpoint inhibition in small cell lung cancer
Source: J Immunother Cancer. 2019 Mar 28;7:87. doi: 10.1186/s40425-019-0572-6 (PMC6437848; doi:10.1186/s40425-019-0572-6)
Supplement: Supplementary file 6 — Figure S6. Kaplan-Meier analysis of progression-free survival (PFS) to first-line chemotherapy in the immunotherapy treated cohort. (DOCX 87 kb) [file 40425_2019_572_MOESM6_ESM.docx]

**Figure S6**

**
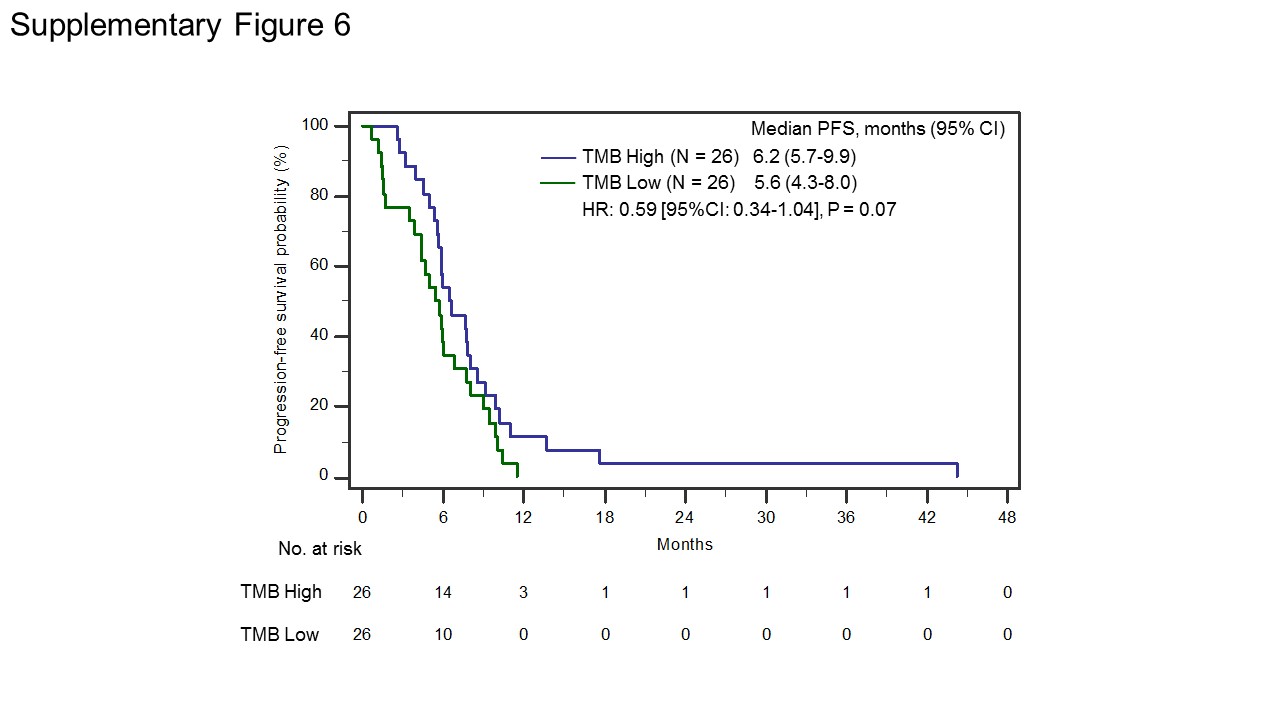
**

**Figure S6.** Kaplan-Meier analysis of progression-free survival (PFS) to first-line chemotherapy in the immunotherapy treated cohort.
